# Risk Perception and Occupational Accidents: A Study of Gas Station Workers in Southern Brazil

**DOI:** 10.3390/ijerph9072362

**Published:** 2012-07-03

**Authors:** Marta Regina Cezar-Vaz, Laurelize Pereira Rocha, Clarice Alves Bonow, Mara Regina Santos da Silva, Joana Cezar Vaz, Letícia Silveira Cardoso

**Affiliations:** 1 School of Nursing, Federal University of Rio Grande, 96201-900 Rio Grande, RS, Brazil; Email: laurelize@gmail.com (L.P.R.); enfcla@yahoo.com.br (C.A.B.); marare@brturbo.com.br (M.R.S.S.); lsc_enf@yahoo.com.br (L.S.C.); 2 School of Chemistry and Food, Oil Refinery Riograndense, 96202-900 Rio Grande, RS, Brazil; Email: joanacezarvaz@yahoo.com.br

**Keywords:** risk perception, occupational accidents, gas station workers

## Abstract

The present study aimed to identify the perceptions of gas station workers about physical, chemical, biological and physiological risk factors to which they are exposed in their work environment; identify types of occupational accidents involving gas station workers and; report the development of a socioenvironmental intervention as a tool for risk communication to gas station workers. A quantitative study was performed with 221 gas station workers in southern Brazil between October and December 2010. Data collection was performed between October to December 2010 via structured interviews. The data were analyzed using SPSS 19.0. The participants identified the following risk types: chemical (93.7%), physical (88.2%), physiological (64.3%) and biological (62.4%). In this sample, 94.1% of gas station workers reported occupational accidents, and 74.2% reported fuel contact with the eyes (*p* < 0.05). It is concluded that workers perceive risks, and that they tend to relate risks with the occurrence of occupational accidents as an indicator of the dangerous nature of their work environment.

## 1. Introduction

This paper discusses the perceptions of gas station workers of the risks they are exposed to in their work environment. It also presents the occurrence of accidents in this environment and the development of a socioenvironmental intervention as a tool for risk communication (RC) to gas station workers.

The motivation for the proposed research came from a literature review about the theoretical approach of risk perception [1,2,3]. On this occasion, the researchers observed the coherence and the need to analyze the issue of human risk in work environments, in the relation with workers, which includes social, cultural and political aspects in its production and reproduction, by workers and by society in general [4,5,6].

According to the theoretical orientation this research assumed, the notion of risk perception involves two factors: the magnitude of the potential loss and the probability of its occurrence [7], in other words, the existence or not of different risk factors and occupational accidents. This might explain why people perceive the same risk in very different situations or why the same individual might perceive risk differently depending on when he or she is asked about it [8]. Risk perception is frequently to be an important factor in risk policy matters [7] in particular companies, as the example in this study, gas stations.

Risk perception encompasses both personal and work environment-related ideas and constructs, because to perceive it, you have to believe it [7]. Therefore, the study of workers’ risk perception is important, as individuals are responsible for the risks perceived in the work environment. The risk an individual perceives might have been caused by that individual. This makes the possibility of changes to minimize or even eliminate risk factors related to individual behavior or even in their own working conditions. One of the processes of interaction to promote the various changes may be the tool of RC.

RC is here understood as an interactive process of exchange of information and opinion among individuals, groups, and institutions [9]. RC can also help promote changes in individual and collective behavior. RC theory and practice may include public participation and conflict resolution. RC, as aforesaid, was used as a tool for the development of speech with an important segment of the gas station work, the workers who supply the vehicles. We emphasize that this feature is one limit of the study, because other segments, in this particular context, will be analyzed in other publications of the authors of this text.

Another theoretical orientation is a classification of different risk factors that the gas station workers are exposed to. Therefore, we used the Act of 16 June 1999 [10] that provides for occupational hygiene and safety standards, and the obligations of employers and employees to create a safe work environment, organization of hygiene and safety at the level of the enterprise, institution and State, procedures for settlement of disputes in this matter, and responsibility for breaches of established standards. In the specific case of gas station employees, during their workday, they are exposed to various occupational risks generated by chemical, physical, biological and physiological risk factors.

Physical risk factors to which workers are exposed on gas station include noise from vehicles, extreme air temperatures (hot and cold) during the seasons with extreme temperatures characteristic of the studied region. Chemical risk factors include contact with the fuel, more specifically, with the chemical benzene. Biological risk factors include bacteria, viruses, fungi, *etc.*, which the gas station workers come into contact due the diversity of clients of the local population and immigrants from other regions, characteristics of the port region of the study, the lack of hand hygiene of workers and non-use of individual protective devices. Physiological risk factors are repetitive movements of the same type, such as those performed by employees of gas station to supply the vehicles. These factors can create or worsen occupational diseases and accidents, which depend on the nature of risk, the degree of exposure, a lack of protective measures, work conditions and rhythms and the worker’s function [11]. An occupational accident is defined as a fire, explosion or another occurrence at work which may endanger the life or health of employees or that of other persons [10].

Gas stations offer favorable conditions for occupational accidents. In this place it is possible to identify health problems related to noise, favoring irritability of the worker; physical stress and decreased hearing acuity [12], among others. Biological agents can result in respiratory infections [13], among others. The inadequate postures, the long working hours standing and repetitive movements may be cause injury and pain in the cervical spine, upper and lower limbs. Please note that the injuries resulting from chemical agents are indicated as a major concern in occupational workplace. Benzene, a constituent of gasoline, is associated with skin lesions and intoxication at the airway and digestive tract levels [14,15,16], myeloblastic and lymphoblastic leukemia and non-Hodgkin lymphoma (NHL) [17,18,19,20]. Chemical hazards are recognized in the literature and in different studies as the risk of greater magnitude and associated with greater potential loss over time, however, it is important to identify the workers’ perceptions of occupational exposure in order to able to understand all risk factors in relation to the their workday.

For these reasons, the present study aimed to identify the perceptions of gas station workers about physical, chemical, biological and physiological risk factors to which they are exposed in the work environment; identify types of occupational accidents involving gas station workers and report the development of a socioenvironmental intervention as a tool for RC to gas station workers.

## 2. Methods

This study consisted of two phases. The first phase was a quantitative, exploratory and descriptive study involving gas station workers, conducted in 2010 in Rio Grande (Rio Grande do Sul, Brazil). The second phase consisted of the report of a socioenvironmental intervention as a tool for RC to workers enrolled in this study, from the results obtained in the exploratory study, conducted in 2011 in the same region.

This study is a part of a larger research project entitled *“Health, Risks and Occupational Diseases: An Integrated Study in Different Work Environments”* [21]. It was approved by the Research Ethics Committee of the Federal University of Rio Grande (Universidade Federal do Rio Grande—FURG). Gas station workers were included in the study after signing an informed consent form. The study was conducted using public funds (National Counsel of Technological and Scientific Development—CNPq) and linked to the Laboratory of Socioenvironmental Process Studies and Collective Production of Health (LAMSA) research group of the Nursing School of the Federal University of Rio Grande.

### 2.1. Subjects

The study subjects were gas station workers in Rio Grande (Rio Grande do Sul, Brazil). Of the 34 gas stations that were invited, 22 agreed to participate. Of the total 340 workers, 221 agreed to participate in the first phase of the study, representing a response rate of 65%. For the second phase, consisting of a socioenvironmental intervention as a tool for RC, all participants of the first phase were asked (221 gas station workers). Although there was insistence for mobilization, nine representatives of three gas stations participated. In addition to the gas station workers, six members of the research group LAMSA also participated, as mediators of the socioenvironmental intervention.

### 2.2. Questionnaire and Data Collection

The first phase of the study was conducted based on the following questions: how gas station workers perceived the risks to which they are exposed in the work environment and which occupational accidents gas stations reported as incurred by them in the work environment? From the theoretical basis assumed in the study, we assumed the existence of a relationship between risk perception and accident involvement by gas station workers. Data collection was performed between October to December 2010, through a structured interview from a questionnaire composed of mixed questions—open, multiple-choice and single-choice. It is noted that for this study, open questions have not been used, which are to be composed from data sources in future publications.

The structured questionnaire had multiple-choice and single-choice questions corresponding with the following variables: participant characteristics (gender, age, skin color/ethnicity, level of schooling and marital status); duration of occupational exposure (duration in the current job and number of work-week hours, which was multiplied by 52, representing the number of weeks in a year); risk perception among workers (the identification of chemical, physical, biological and physiological risk factors); and the occurrence of occupational accidents self-reported by workers.

For organization of socioenvironmental intervention, we began with the characterization of the research subjects (structured questionnaire) and scientific research in the literature to support the targeted intervention to the risks in the gas station work environment.

The research was structured primarily based on Diseases Work-Related Manual, U.S. EPA. Toxicological Review of Benzene, Act of 16 June 1999, and IARC Monographs on the evaluation of carcinogenic risks to humans and the Prevention Report of RC [9,10,11,22,23]. These documents include aspects of the health surveillance of workers exposed to different risk factors in their work environment and the prevention of exposure to these factors.

After analysis of this data, we decided to structure a plan for a Production Health Workshop, which included the topics: occupational risk generated by chemical, physical, biological and physiological factors; risk perception and occupational accidents, prevention of accidents or health problems. To work out these issues, we focused on the approach of the theme perception of risk factors (physical, chemical, biological and physiological) to which gas station workers are exposed and accidents arising from the characteristics of the work done by them, through discussion with workers about Personal Protective Devices (PPD) that could minimize the risk exposure of the work environment risks and possible strategies identified by workers to minimize work risks.

To finalize the socioenvironmental intervention workers were offered a coffee-break with orange juice, water, biscuit and sandwiches. These foods served to emphasize the dialogue on healthy eating with a focus on increasing the natural body’s defenses.

### 2.3. Data Analysis

The questionnaire’s reliability was assessed with Cronbach’s alpha, which was 0.96, indicating that variance in the scores is explainable. The Statistical Package for Social Sciences (SPSS) software Version 19.0 was used to organize and analyze the data. We started by descriptive analysis and further inferential analysis was performed. We used Fisher’s exact test to verify the existence of an association between perceived risk and occurrence of accidents. Analyzes the influence of exposure time on the occurrence of occupational accidents and worker’s risk perception were performed using Mann-Whitney test.

## 3. Results

### 3.1. Participant Characteristics

The sample included 221 workers from 22 (65%) gas stations in a southern Brazilian town. Most workers (200; 90.5%) were male; 189 (85.9%) were ethnically white; and 115 (52%) were single. Their ages ranged from 19 to 64 years, with an average of 30.25 years (±9.58), and 111 (50.2%) had finished secondary school (Table 1).

**Table 1:** Demographic characteristics of study subjects (n = 221) *.

Variables	Categories	n	%
Gender	Male	200	90.5
	Female	21	9.5
Marital status	Single	115	52
	Married	97	43.9
	Separated	9	4.1
Skin color/ethnicity	White	189	85.5
	Black	24	10.8
	Asian	5	2.3
	Indigenous	2	0.9
	Not known	1	0.5
Schooling *	Elementary school, incomplete	29	13.1
	Elementary school	8	17.2
	Secondary school, incomplete	32	14.5
	Secondary school	111	50.2
	Higher education, incomplete	8	3.6
	Higher education	1	0.5
	Postgraduate education	1	0.5
	Not answered	1	0.5

* Numbers for each item may total less than total n’s because of missing values.

### 3.2. Risk Perception

The results of the questionnaire on risk perception in the work environment showed that 207 (93.7%) workers identified chemical risks, 195 (88.2%) identified physical risks, 142 (64.3%) identified physiological risks and 138 (62.4%) identified biological risks. Among the risk factors identified, the most frequent was contact with chemical products in the workplace, cited by 176 (79.6%) workers (Table 2).

**Table 2:** Perception of gas station workers about physical, chemical, biological and physiological risk factors (n = 221) *.

Risk factors	n	Percent (%)
Physical		
Cold	162	73.3
Moisture	150	67.9
Noise	125	56.6
Heat	120	54.3
Vibrations	47	21.3
Non-ionizing radiation	27	12.2
Ionizing radiation	17	7.7
Abnormal pressures	17	7.7
**Chemicals**		
Chemical products	176	79.6
Dust	159	71.9
Gases	155	70.1
Vapors	131	59.3
Mist	106	48.0
Fumes	82	37.1
Fog	70	31.7
**Biological**		
Bacteria	115	52.0
Virus	110	49.8
Fungi	60	27.1
Protozoa	39	17.6
Parasites	35	15.8
Bacilli	28	12.7
**Physiological**		
Poor posture	83	37.6
Repetitive strain	83	37.6
Slippery	39	17.6
Inadequate lighting	29	13.1
Lifting heavy loads	25	11.3
Materials scattered on the floor	10	4.5

* Numbers for each item may total less than total n’s because of missing values.

The self-reported risks were correlated with the duration on the job, and higher rates of risk perception were found among individuals with lower work durations (Table 3). These results can be explained by the greater number of workers (71) that fell into the categories of zero to five years of occupational exposure and 40 to 50 weekly work hours. The self-reported risks were adjusted for the duration of occupational exposure (duration at the gas station job × work-week hours) and showed no significant differences.

**Table 3:** Risk factors and time of exposition reported by workers (n = 221) *.

Risks	Duration on the job (years)	Weekly work hours	Yes (n)	%	No (n)	%
Chemical	0–5	30–40	59	89.4	07	10.6
		40–50	69	97.2	02	2.8
		>50	16	100.0	00	0.0
	5–10	30–40	09	100.0	00	0.0
		40–50	16	100.0	00	0.0
		>50	02	100.0	00	0.0
	>10	30–40	15	83.3	03	16.7
		40–50	14	87.5	02	12.5
		>50	06	100.0	00	0.0
Physical	0–5	30–40	56	84.8	10	15.2
		40–50	64	90.1	07	9.9
		>50	13	81.3	03	18.8
	5–10	30–40	08	88.9	01	11.1
		40–50	16	100.0	00	0.0
		>50	01	50.0	01	50.0
	>10	30–40	15	83.3	03	16.7
		40–50	15	93.8	01	6.3
		>50	06	100.0	00	0.0
Physiological	0–5	30–40	40	60.6	26	39.4
		40–50	52	73.2	19	26.8
		>50	11	68.8	05	31.3
	5–10	30–40	07	77.8	02	22.2
		40–50	08	50.0	08	50.0
		>50	01	50.0	01	50.0
	>10	30-40	11	61.1	07	38.9
		40–50	09	56.3	07	43.8
		>50	02	33.3	04	66.7
Biological	0–5	30–40	34	51.5	32	48.5
		40–50	49	69.0	22	31.0
		>50	10	62.5	06	37.5
	5–10	30–40	05	55.6	04	44.4
		40–50	13	81.3	03	18.8
		>50	02	100.0	00	0.0
	>10	30–40	11	61.1	07	38.9
		40–50	10	62.5	06	37.5
		>50	03	50.0	03	50.0

* Numbers for each item may sum less than the total n values because of missing values.

### 3.3. Occupational Accidents

Occupational accidents were reported by 94.1% (208) of the participants (Table 4). The most frequently reported occupational accidents reported was contact between fuel and skin, which was reported by 202 workers (91.4%).

**Table 4:** Occupational accidents reported by gas station workers (n = 221) *.

Occupational accidents	n	Percent (%)
Fuel leak	163	73.8
Skin contact with fuel (gasoline, alcohol, diesel)	202	91.4
Outpouring of fuel (gasoline, diesel) on the worker	138	62.4
Eye contact with fuel (gasoline, alcohol, diesel)	164	74.2
Contact f another substances (detergent, grease, dust) in the eyes	118	53.4
Fuel inhalation	172	77.8
Collision between car and workers	178	80.5

* Numbers for each item may total less than total n’s because of missing values.

The self-reported occupational accidents were adjusted for duration of exposure and showed a significant difference (*p* = 0.012) for contact of fuel with the eyes. For those with longer exposure to fuel, the risk for this type of occupational accident was greater.

The self-reported (chemical, physical, ergonomic and biological) risk factors were adjusted for the occurrence of occupational accidents per risk type and the full set of risks. In the first case, the chemical and biological risks exhibited significant differences (*p* = 0.05), as did the full set of risks grouped together (*p* = 0.029).

### 3.4. Socioenvironmental Intervention with Gas Station Workers

The socioenvironmental intervention was attended by nine gas station workers and six researchers from LAMSA. The time used for planning was 40 hours and that to perform the intervention during the Production Health Workshop was four hours. We note that one of the obvious limitations of the intervention process was the low number of participants (n = 9). Besides the numerical limit, the socioenvironmental intervention was developed without participation of the workforce managers. Under these circumstances, we consider this as a pilot socioenvironmental intervention for RC which could be repeated with other groups of workers exposed to similar working conditions. Also, as mentioned this practice included the Health Production in Different Work Environments Program (HPDWEP), of LAMSA, School Nursing, Federal University of Rio Grande/Brazil. The HPDWEP consists of a set coordinated actions and continuous shaft in promoting social and environmental health in different work environments, whose environments are included in the study group’s academic LAMSA. The debate during the socioenvironmental intervention is summarized in Table 5 and Table 6.

The socioenvironmental intervention was developed based on the RC concept [1,2,3,24]. We developed content (message) about the nature of risk, through the classification of risk factors (physical, chemical, biological and physiological) and occupational health and safety legislation of the Brazilian Ministry of Health, the Occupational Safety and Health Act of 16 June 1999 of International Labour Organization (ILO).

**Table 5:** Personal Protective Devices reported by gas station workers that could minimize the workplace risks (n = 9) *.

Individual Protective Devices	n	Percent (%)
Gloves	7	77.7
Apron	2	22.2
Mask	9	100
Boots	3	33.3
Working clothes	3	33.3
Safety glasses	2	22.2

* Numbers for each item may total less than total n’s because of missing values.

**Table 6:** Strategies identified by gas station workers that can be taken to minimize risk in the workplace (n = 9) *.

Strategies for minimizing risks	n	Percent (%)
Avoid contact with fuel	2	22.2
Wash hands regularly	1	11.1
Drinking water	1	11.1
Exercising regularly	2	22.2
Conducting workshops with managers	2	22.2
Use of PPD	4	44.4
Disclosure of risks at gas stations	1	11.1
Health care for workers about food	1	11.1

* Numbers for each item may total less than total n’s because of missing values.

To trigger the development of communication (first step) with the workers who are participating of the intervention, we used the question: What are the risks that you identify in your workplace? We asked that workers imagine their workplace to respond to the question. The responses were expressed in a mural with colors corresponding to the classification of risk factors. This allowed the demonstration of workers through comparisons, considerations and suggestions of the theme. Visualization of risk factors from the colors allowed us to show the predominance of chemical risk factors as the main theme, to continue the RC process. We noted that this was corroborated in other studies as a risk factor of greatest potential hazard to workers’ health in the long term [14,15,16,17,18,19,20,21,25,26,27,28,21,25].

To continue the communication, we presented the results of this research (second step). This approach focused on returning the perception of risk factors (physical, chemical, biological and physiological) to which workers are exposed on gas station and accidents arising from the characteristics of their work. Corroborating the findings in the study, references were made by workers who participated in the socioenvironmental intervention, particularly the chemical risk factor. We understand this fact, because this is the most prevalent agent (raw material) the gas station workers handle in their daily work. Thus, we used the perception of chemical risk factors, such as a concrete reality of the gas station workers, to develop the sequence of intervention.

Communicate continued with a discussion on preventive measures regarding the use of PPD and healthy eating (third step). The debate on this theme is summarized in Table 5 and Table 6. Included to strengthen the interactive process and exchange (RC), a coffee-break with orange juice, water, fruits and sandwiches was provided, for the closure of an argumentative dialogue about healthy eating with a focus on increasing the body’s natural defenses.

We concluded with an assessment, in which it was possible identify the importance of socioenvironmental intervention for workers’ health who participated before, because the reporting of workers on the lack of information about workplace risks and reflection of new attitudes about risk prevention, accidents and injuries to health. We provided an explanatory poster about health protective measures, for each participating worker so they could display it at the workplace and encourage their colleagues to implement protective measures to minimize exposure to risk factors.

## 4. Discussion

This study contributes to an understanding of the perception of risk factors and the occurrence of occupational accidents among gas station workers. As regards the perception of risk factors that were identified that reported risks in decreasing order are: chemical, physical, physiological and biological. Regarding accidents occurring to gas station workers, we found that the accidental skin contact with fuel (gasoline, alcohol, diesel) was the most frequently mentioned (91.4%) one and inhalation of fuel was also reported (77.8%). The gas station workers are continually in contact with fuel, depending on the activities they do. They are even more centralized in handling several liters of fuel (gasoline, alcohol, diesel) and constant inhalation of vapors emitted by vehicles, which can lead to a greater number of accidents of this nature [14,15,16]. It must also consider that these workers face different types of vehicles. Depending on the type of vehicle, the fuel exposure can be larger or smaller. The greater the fuel exposure, the greater is the chance of contact with skin and eyes of the worker with the fuel. Moreover, the need of workers to ‘sniff’ the tank cap to ensure the type of fuel contained in order to avoid mistakes causes fuel inhalation [12].

The sample in the present study was composed mostly of young, white, male adults, which is consistent with the samples used in other reports [29,30]. The fact that most participants were male might be related to occupations involving obvious risks, such as truck drivers, port workers, and workers exposed to benzene, usually being filled by males.

The findings also suggest that the perception of chemical risk and the occurrence of accidents involving this risk were present more frequently. This risk perception related to the chemical risk and chemical occupational accident, is due to the raw material that the worker handle in their daily work, for example, gasoline [31]. Gasoline is derived from oil and composed of aromatic hydrocarbons, including benzene, toluene and xylene (BTX) [15]. Benzene is an important chemical because its physical properties can be modified as a function of vapor pressure, resulting in the production of dangerous toxic gases [31]. According to the classification system developed by the International Agency for Research on Cancer (IARC) [23], benzene belongs to Group 1, which comprises compounds or physical factors carcinogenic for humans. Therefore, special attention must be paid to the degree and duration of benzene exposure because safe levels of benzene are uncertain and depend on other factors, such as absorption susceptibility [31,32].

Regarding chemical risk, 20% of the investigated workers identified chemicals used in their work environment, 17% identified gases derived from fuels, and 14% identified the vapors emitted by cars, for a total of 51% of the sample. In other studies [16,17,31], the frequent inhalation of vapors emitted by cars, direct handling of gas pumps and daily exposure to several liters of fuels were indicated as the main factors of exposure.

This study documented and reported that chemical occupational accidents are frequent among gas station workers that have longer exposure times. In addition, in this study, the exposure time was higher in the case of ‘eye contact with fuel’ chemical occupational accidents. In this occupational accident example, it is known that the absorption capacity of the agent benzene can be increased by contact with the mucosa of the eye and the mouth [33,34]. Skin contact with fuel was reported by 91.4% of gas station workers, and it is known that there is a potential way for absorption, because of the ability to the fuel (liquid phase or vapor) to permeate the skin, small latency contact and high toxicity, even after brief exposure. 

Some studies [25,26,27] have demonstrated benzene’s myelotoxic potential, which is manifested by a decreased number of leukocytes (both granulocytes and lymphocytes) and platelets, lower hemoglobin concentration, and fewer progenitor myeloid cells in workers exposed to levels ≤1 ppm (parts per million), which is the exposure level of gas station workers. Although it was not the object of the present study, it is stated that the occupational accident contact with fuel exposes workers in our study to the benzene’s myelotoxic potential.

Another study [16] showed that gas station workers are at high risk of chemical risk factor exposure as a function of the work environment, tasks performed and products handled daily. These circumstances make it difficult to avoid occupational exposure to chemicals. However, other studies [35] have suggested protective measures that might reduce the risk exposure and occupational accidents: using personal and collective protection equipment, changing clothes for each shift and applying hygiene measures, such as hand washing.

The present study represents a contribution to the occupational health policy formulated by the Permanent National Commission on Benzene [32], which supervises the implementation and development of the Benzene Agreement, which aims to prevent occupational exposure to this chemical and thus, protect workers’ health. It is believed that understanding risk perception in the work environment as a socioenvironmental health phenomenon will contribute to preventive measures that reduce the occurrence of occupational accidents. For this purpose, workers must learn about risks, their consequences and how to prevent them.

In addition, Regulating Norm #16, a piece of Brazilian legislation backed by the Ministry of Work and Employment, addresses dangerous activities and operations involving combustible materials, and its Appendix 2 grants a 30% increase in the salaries of workers operating pumps with inflammable liquid fuels and to other gas station workers due to the dangers inherent to their work environment [36]. Another study [37] on the perception of chemical risks showed that although workers do perceive the existence of risk, they are not continually concerned with this aspect of their jobs. This scenario might be explained by the fact that living with a continual awareness of risks would be unbearable. The authors also hypothesized that several risks inherent to work become acceptable over time, and/or that occasionally, workers have no option but to accept the risk. This applies even when the chemical risk perception is based on sensations felt by workers or their colleagues.

Additionally, physical risk was identified and reported by 88.2% of the present sample, which agrees with another study of gas station workers [13]. The workers in that study reported feeling unsafe due to their daily exposure to climactic changes. For gas station workers, physical risks represent the discomforts caused by the work environment itself.

Physiological risk was self-reported by 64.3% of workers, who most frequently identified inappropriate posture and repetitive stress. These findings agree with those of another study [12], in which gas station workers’ main health complaint was the need to stay in a standing position all day. This perception by the workers might be attributed to the situations they experience [37].

Biological risk was identified by 62.4% of workers. The workers indicated that the main contaminating microorganisms were bacteria and viruses, which might be related to their frequent contact with customers, inappropriate hygiene conditions in the work environment and insufficient measures for individual protection [13,16,35]. Regarding biological risk perception, one study [38] identified the workers’ perception of imminent infectious disease risk. The results showed that risk perception varied as a function of the frequency of the workers’ exposure to contaminated fluids, knowledge of customers’ diseases and history of previous accidents [7].

In the present study, the correlation between chemical and biological factors and occupational accidents was statistically significant. This finding agrees with another study [13] performed with gas station workers in which most workers with longer duration on the job identified a lack of physical safety as a risk associated with robberies, explosions and being run over by vehicles. This finding might be related to a lack of information about the types of risk that can cause long-term damage, such as chemical intoxication or risk perception that causes immediate damage. The relationship between duration of exposure and occupational accidents might be explained by the following observation [39]: the more time a worker spends near gas pumps or fuel, the greater the exposure.

From the presented exploratory study, there was a socioenvironmental intervention for gas station workers. The socioenvironmental intervention in the work encourages workers to think about the risk factors in the workplace that can cause illness or accidents. In the specific case of the socioenvironmental intervention described in this study, means were used to encourage workers to visualize ways of minimizing risk factors and, therefore, occupational accidents, is the case of PPD that can be used in the workplace and visualization of strategies for minimization of risk factors. The results of the socioenvironmental intervention indicate that PPD and strategies for minimizing the risk factors reported by gas station workers indicated the concern of workers with chemical risk factor, which confirms the results of the exploratory study.

The socioenvironmental intervention performed in this study applied strategies that stimulated workers to minimize individual and collective risks, because it is a process in which workers seize and multiply the knowledge in their work environment, and thus interfering with the collective work conditions.

A horizontal intervention technique was applied via a dialogue (communication) between researchers and workers in which the researchers offered spontaneous narratives and posed questions. This experience shows that promoting socioenvironmental interventions facilitates the use of clinical reasoning based on theoretical-scientific elements to help workers: (1) correlate theoretical features with their own empirical observations on the risks present in their environment, (2) reflect and (3) perceive possible prevention measures.

Some studies have indicated that workers’ participation in decision making is of paramount importance to their safety and health [37,40,41]. Workers want to be consulted about their own needs and do not object to data collection for assessing high-risk procedures when they are duly warned about the potential risks of the materials they work with [37]. For this reason, workers’ risk perception must be considered when applying strategies for changing work environments [40]. By performing socioenvironmental health-based interventions, nurses might motivate workers to seek such changes. However, the decision to adopt these health-related orientations is exclusively the prerogative of the workers themselves.

## 5. Conclusions

In conclusion, gas station workers realize they are exposed to risk factors, especially chemical risk factors due to their workplace being particularly dangerous. The frequency of occupational accidents tends to a state that allows the perception of risk factors to be realized from the accident to the worker.

Such evidence confirms the findings of literature on risk factors that gas station workers face in the workplace and in similar situations to those found in this particular study. This study highlights the importance of review and changes in work conditions at gas stations (for example, avoiding skin contact with fuel with workers using gloves), like other realities that already exceed the unsanitary conditions. To appropriately address the needs of specific worker subgroups, occupational health professionals must grapple with the complexity that exists within the workforce.

We believe that the RC is a participatory process designed to assist individuals or groups to make decisions that will advance their health and well being. Health promotion theorists define empowerment as a multidimensional construct that attends to individual, small group of workers and organizational of health promotion. The strategy of RC constitutes a positive possibility of learning about risk factors and the individual and collective measures to minimize the accidents. Occupational health professionals play an important role in responding to the unique needs of individuals and subgroups that make up a diverse workforce by targeting subgroups with health promotional campaigns and advocating for healthy workplaces. 

We understand that knowing about workers’ perception about a particular set of occupational hazards, is essential to prepare an effective plan for RC. Thus, our intervention has limits, but is included in HPDWEP. Therefore, the socioenvironmental intervention for RC, was confirmed as a positive strategy to promote the socioenvironmental health of workers in their workplace.
